# FindZebra - the search engine for difficult medical cases

**DOI:** 10.1186/1750-1172-9-S1-O5

**Published:** 2014-11-11

**Authors:** Ole Winther, Dan Svenstrup, Philip P Henningsen, Róbert Kristiásson, Henrik L Jørgensen

**Affiliations:** 1 Department for Applied Mathematics and Computer Science, Technical University of Denmark (DTU), 2800 Lyngby, Denmark; 2 Copenhagen University Hospital, Department of Clinical Biochemistry, Bispebjerg Hospital, Copenhagen, Denmark

## 

Physicians as well as laypeople use both general purpose and specialised web-based search tools when confronted with medical questions. FindZebra.com is a tool for generating hypotheses about rare disease diagnosis. It uses freely available high quality curated information on rare diseases and open source information retrieval software (Apache Lucene Solr) tailored to the problem. FindZebra is intended primarily for physicians and other professionals concerned with diagnosis of rare diseases. Our benchmarking against several search tools (Google search, PubMed and http://omim.org) shows that FindZebra has higher performance in retrieving the correct diagnosis when queried with symptoms [1,2]. Our findings indicate that the ranking algorithm used is the most important factor for the success.

## References

[B1] Dragusin Radu Petcu Paula Lioma Christina Larsen Birger Jørgensen Henrik L Cox Ingemar Hansen Lars K Ingwersen Peter Winther Ole FindZebra: a Search Engine for Rare Diseases International Journal of Medical Informatics, IJMI 2013 23462700 10.1016/j.ijmedinf.2013.01.005

[B2] Dragusin Radu Petcu Paula Lioma Christina Larsen Birger Jørgensen Henrik L Cox Ingemar Hansen Lars K Ingwersen Peter Winther Ole Specialised tools are needed when searching the web for rare disease diagnoses Rare Diseases 2013 25002998 10.4161/rdis.25001 PMC3932942

